# Machine learning detection of epileptic seizure onset zone from iEEG

**DOI:** 10.1007/s13534-025-00480-w

**Published:** 2025-05-27

**Authors:** Nawara Mahmood Broti, Masaki Iwasaki, Yumie Ono

**Affiliations:** 1 Electrical Engineering Program, Graduate School of Science and Technology, Meiji University, Kawasaki, Kanagawa Japan; 2 Department of Neurosurgery, National Center Hospital, National Center of Neurology and Psychiatry, Kodaira, Tokyo Japan; 3 Department of Electronics and Bioinformatics, School of Science and Technology, Meiji University, 1-1-1 Higashi-Mita, Tama-ku,, Kawasaki, Kanagawa Japan

## Abstract

Accurate identification of seizure onset zones (SOZ) is essential for the surgical treatment of epilepsy. This narrative review examines recent advances in machine learning approaches for SOZ localization using intracranial electroencephalography (iEEG) data. Existing studies are analyzed while addressing key questions: What machine learning techniques are used for SOZ localization? How effective are these methods? What are the limitations, and what solutions can drive further progress in the field? This narrative review examined peer-reviewed studies that employed machine learning techniques for SOZ localization using iEEG data. The selected studies were analyzed to identify trends in machine learning applications, performance metrics, benefits, and challenges associated with SOZ identification. The review highlights the increasing adoption of machine learning for SOZ localization, mostly with supervised approaches. Particularly support vector machine (SVM) using high frequency oscillation (HFO) biomarker feature being the most prevalent. High accuracy and sensitivity, especially in studies with smaller sample sizes are reported. However, patient-wise validation reveals limited generalizability. Additionally, ambiguity in SOZ definition and the scarcity of open-access iEEG datasets continue to hinder progress and reproducibility in the field. Machine learning offers significant potential for advancing SOZ localization. Development of more robust algorithms, integration of multimodal data, and greater model interpretability, can improve model reliability, ensure consistency, and enhance real-world applicability, thereby transforming the future of SOZ localization.

## Introduction

Epilepsy is a neurological disorder characterized by recurrent, unprovoked seizures resulting from abnormal electrical activity in the brain [1]. It affects people of all ages and impacts approximately 70 million individuals worldwide [2, 3]. While many patients achieve seizure control with medication, nearly one-third of the patients experience drug-resistant epilepsy, when appropriately selected medications fail to control seizures [4–6]. As treatment, surgical intervention is performed to resect or ablate epileptogenic brain tissue containing seizure foci [6]. Success in such surgeries relies heavily on accurately identifying the region or neural network in the brain from where seizure originates, which is usually defined as seizure onset zone (SOZ) [7, 8]. However, the SOZ is often located near critical brain regions responsible for essential functions such as vision, language, and movement, making its identification and resection highly complex [10, 11].

Traditionally, neurologists identify SOZ by visually assessing electroencephalography (EEG) [9], supplemented by the findings from seizure symptoms recorded during long-term video EEG monitoring [12] and from neuroimaging modalities such as magnetic resonance imaging (MRI) [14], positron emission tomography (PET) [15], single‑photon emission computed tomography (SPECT) [15] and magnetoencephalography (MEG) [13]. Among these clinical methods, intracranial EEG (iEEG) is considered the gold standard for guiding surgical planning in patients [5, 16]. iEEG data provides high resolution and continuous signals (signals typically span from 0.1 Hz to 500 Hz) [17]. However, manual inspection, marking, and SOZ identification are time-consuming, expertise-dependent tasks that require intensive and collaborative discussion among clinicians [18, 19]. Due to the infrequency of epileptic activity, long-duration recordings are required, making manual inspection complex and data collection invasive for patients. To address these challenges, researchers are developing automated methods for identifying epileptic biomarkers and localizing the SOZ, offering faster and more accurate support for treatment planning in drug-resistant epilepsy [5, 19].

Extensive research has focused on automated methods to detect seizures, biomarkers, and SOZs using EEG/iEEG data [5, 19–21]. Leveraging signal processing, machine learning, and deep learning, these systems aim to improve SOZ localization and support clinical decision-making [22, 23]. With the growing role of automation in epilepsy treatment, reviews are essential to consolidate findings, highlight challenges, and guide future research. They bridge clinical needs with technological advances and help assess the clinical applicability of emerging machine learning approaches. Previous reviews have addressed key aspects of SOZ localization. Hussein et al. [24] provided an in-depth analysis of feature and classifier selection for focal and non-focal channels. Islam et al. [25] focused on artificial intelligence based methods using iEEG signals for seizure focus detection. Balaji et al. [5] reviewed iEEG approaches for identifying biomarkers related to SOZ, while Mahnoosh et al. [19] explored integrating EEG and neuroimaging with machine learning to enhance detection. While these reviews have advanced our understanding of automation in the field of SOZ, there is a need for an updated narrative that critically discusses the advantages and challenges of applying machine learning to SOZ identification.

This narrative review distinguishes itself from existing literature through its exclusive focus on machine learning based SOZ localization from iEEG signal. This study incorporates a detailed discussion on algorithmic trends, success points, and weaknesses specifically in SOZ identification, which are often overlooked or vaguely discussed in other reviews. By critically analyzing existing research, this review aims to help identify up-to-date trends and highlight avenues for future improvement, aiding technological innovation to align with clinical needs. To this end, we tried to find the answers to the following questions.

- What machine learning approaches are currently utilized to localize the SOZ in the EEG signals?
- How did these approaches perform, and what were their merits?
- What limitations are still there in applying machine learning in SOZ localization? What are the possible solutions?

The remainder of this paper is organized as follows. Section 2 explains the basics of iEEG and its relevance to SOZ. The methodology followed in this review is explained in Sect. 3. Section 4 illustrates the trend in implementing machine learning in SOZ identification. Section 5 identifies the advantages of using machine learning in SOZ localization, while Sect. 6 discusses the shortcomings of the existing approaches and possible solutions. Section 7 discusses the overall impact of the review and sheds light on future aspects of this research field, followed by the conclusions drawn from the review presented in Sect. 8.

## Background

**Fig. 1:**
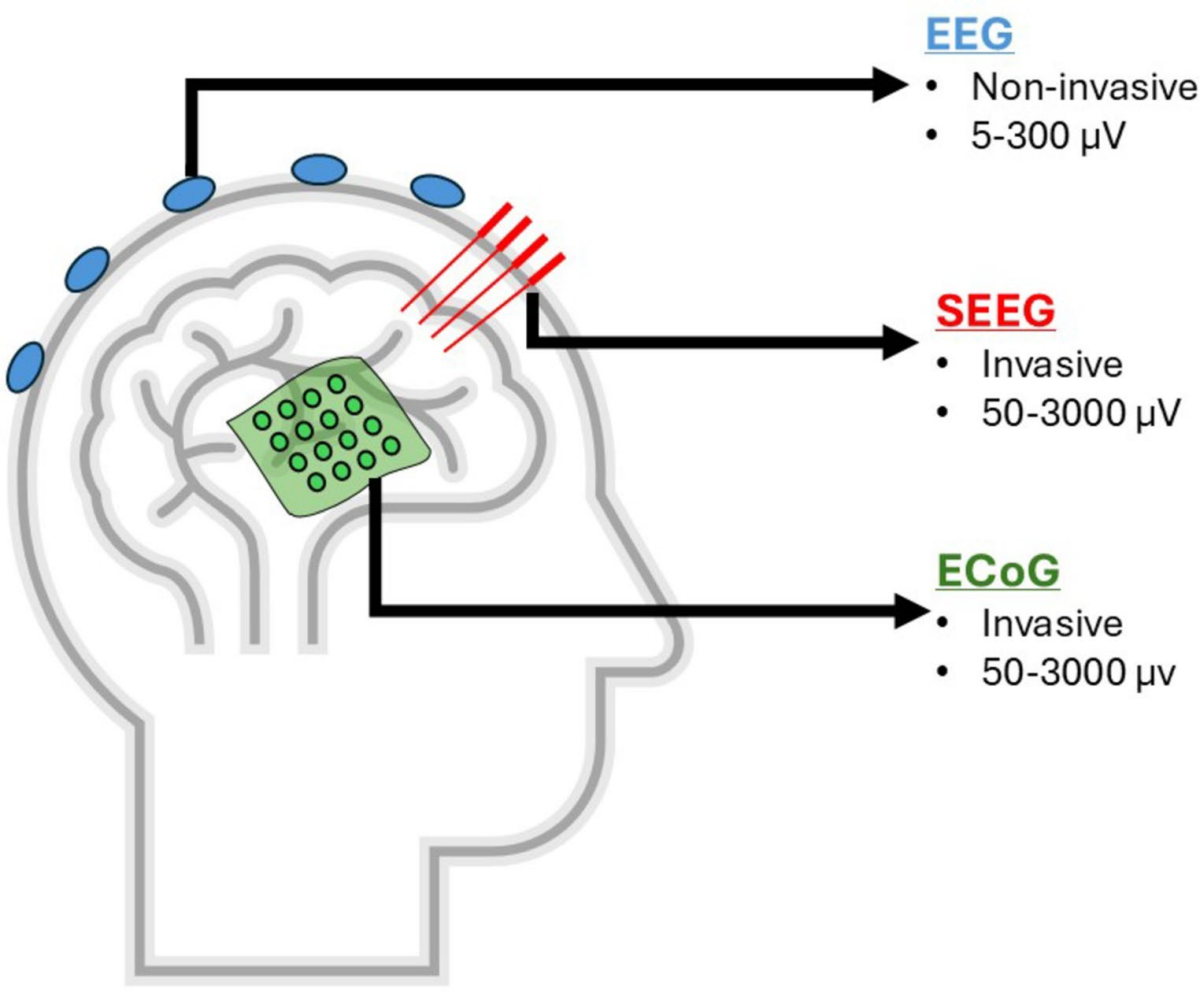
Methods for recording brain activity: EEG, SEEG, and ECoG. Each method has distinct characteristics in terms of invasiveness and signal amplitude range

Seizure onset is characterized by a sudden, distinct change in neural activity, marked by alterations in both frequency and amplitude, differentiating it from the background activity [26]. The region of the brain’s cortex where seizures originate is commonly known as the epileptic seizure focus, often conceptually described as the SOZ [27]. High-resolution, noise-free data significantly enhances the precision of SOZ boundary localization. iEEG is a form of electroencephalography in which electrodes are positioned directly on or within the cortex to capture electrical activity [28]. Electrocorticography (ECoG) and Stereoelectroencephalography (SEEG) are two iEEG techniques capable of simultaneously recording neocortical activity using tens to hundreds of electrodes [29]. ECoG is measured using sheet electrodes placed on the cortical surface, while SEEG is measured by inserting rod-shaped multi-point electrodes through small holes drilled into the skull. Unlike traditional EEG, which collects signals via the skull, iEEG gives higher spatial and temporal resolution data with minimal interference, making it effective, especially in pinpointing specific brain regions linked to neurological problems (Fig. 1). For this reason, when it comes to locating SOZ, iEEG has been considered as an indispensable source of data in certain situations [5, 16].

iEEG signal can be categorized as either ictal or interictal, depending on when it’s recorded. Ictal data is captured during a seizure and reflects the abnormal, synchronized brain activity characteristic of the seizure itself. Interictal data, on the other hand, is recorded between seizures. While no seizure activity is present during this period, interictal data still contains abnormal electrical activities that can indicate SOZ location. Furthermore, iEEG recordings can capture neural activity in the brain and are typically categorized into different frequency bands: Delta (up to 4 Hz), Theta (4–8 Hz), Alpha (8–133 Hz), Beta (13–30 Hz), Gamma (30–150 Hz), Ripple (150–250 Hz), and Fast Ripple (above 250 Hz) [5, 39]. During presurgical evaluations, the SOZ is clinically localized by analyzing different frequency band iEEG recordings from individual electrodes. This process involves features extracted from the raw iEEG signal across various domains, including temporal, spatial, and frequency domains. Features may include amplitude, pattern, waveform, event, or entropy, which are mostly found in signals from the ictal period [30–38]. Furthermore, SOZ is often identified using unique features or signatures in brain activity, also known as epilepsy biomarkers. Several types of epilepsy biomarkers can be identified during both ictal and interictal periods and within different frequency ranges. Some common ones are:

- *High-Frequency Oscillations (HFOs)*: HFOs are transient brain activities in the ripple and fast ripple band of iEEG (80–500 Hz frequency range). Key parameters such as HFO rate, amplitude, duration, spectrum, repetition and power are commonly analyzed to assess their potential as biomarkers for the SOZ [4, 6, 11, 40–43,74 ]. HFOs can occur during both ictal and interictal periods. While studies have shown that HFOs can be more frequent during the ictal period compared to the interictal period [6], HFO features are still widely extracted from interictal data in many studies [55–58].
- *Interictal epileptiform discharges (IEDs)*: IEDs are abnormal, spike-like electrical discharges that occur between seizures, during the interictal period. IEDs are characterized by their distinctive shape and waveform, which typically include a sharp wave followed by a slow wave and found in lower frequency bands (e.g. theta, alpha, beta). IED rate, density, or spike burst are potential indicators of SOZ [11, 44, 45].
- *Causal connectivity*: Causal connectivity describes the directed flow of alterations in information and causal interactions between brain regions, calculated by partial directed coherence or Granger causality [46]. It can capture how the influence between brain regions changes over time. The directional information, spectra, and net flow calculated from causal connectivity measures during interictal period have proven helpful in localizing the SOZ across a range of frequency bands, including both high and low frequencies [47–49].
- *Phase‑amplitude coupling (PAC)*: PAC refers to the interaction between different brain rhythms, where the phase of a lower-frequency oscillation (such as delta, alpha, beta band) modulates the amplitude of a higher-frequency oscillation (such as gamma, ripple). It is a form of cross-frequency coupling that reveals interactions between brain frequencies. PAC strength, intensity, and pattern are widely studied as a marker for neurological conditions like epilepsy [45, 50–52].

**Fig. 2:**
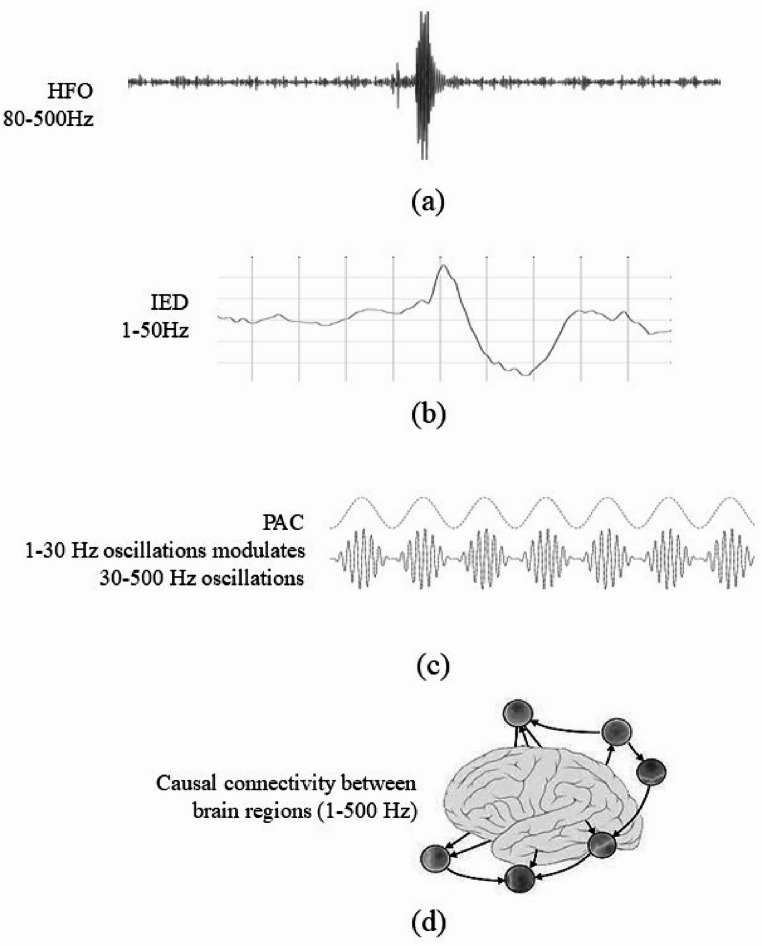
Illustration of key electrophysiological biomarkers for SOZ localization. (a) HFOs: found in the 80–500 Hz range. (b) IEDs, found in 1–50 Hz range. (c) PAC, where low-frequency oscillations (1–30 Hz) modulate the amplitude of high-frequency oscillations (30–500 Hz). (d) Causal connectivity between brain regions, representing directed interactions in the 1–500 Hz range

Figure 2. Illustrates a visualization of these key biomarkers along with their frequency range.

## Methodology

**Fig. 3:**
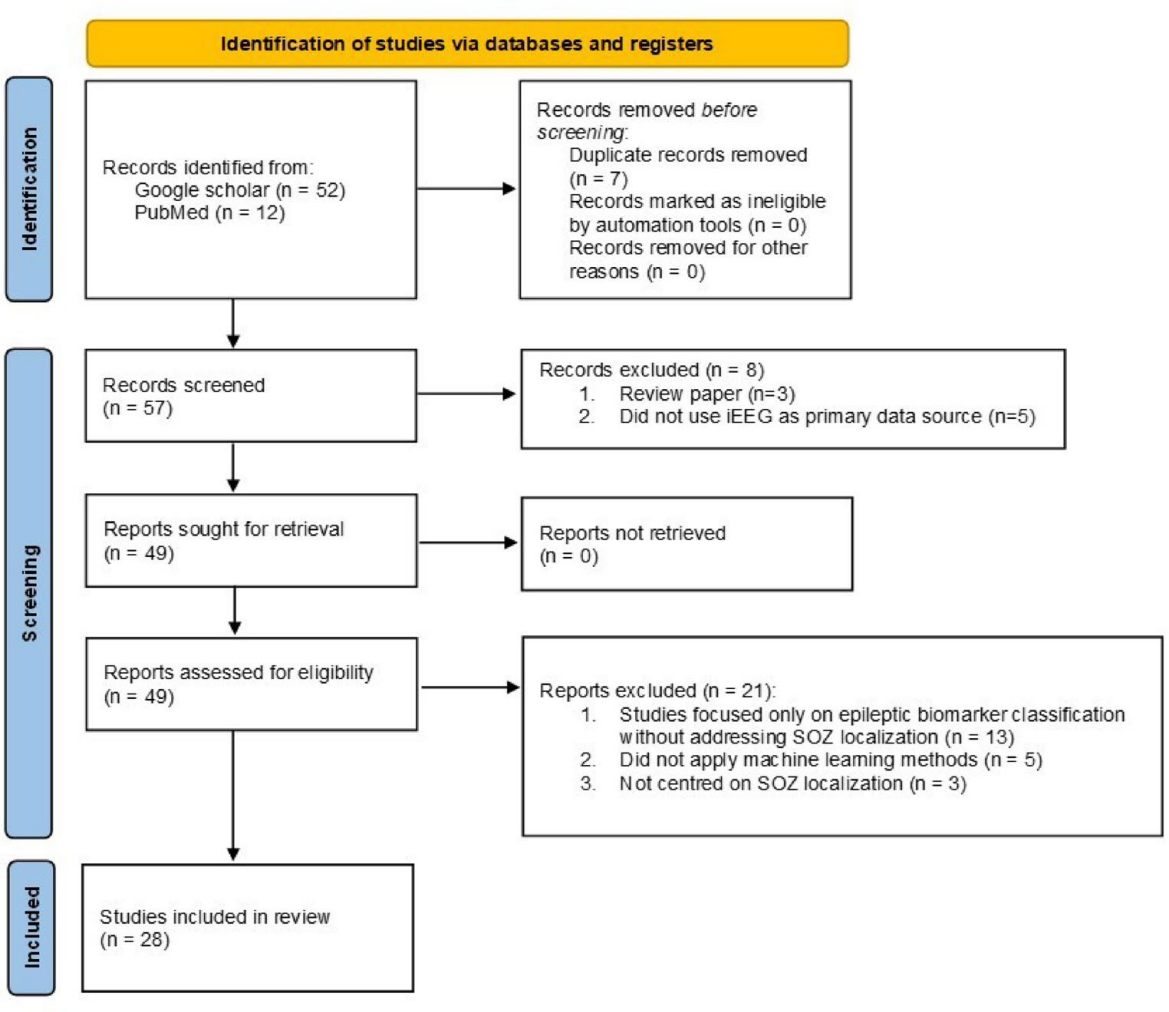
The flow diagram illustrating the systematic screening and selection process

This narrative review aims to synthesize recent advances in SOZ localization and classification using iEEG and machine learning techniques. Although this is a narrative review, we followed a structured approach inspired by the PRISMA guidelines to enhance transparency and reproducibility. A systematic search and screening process was conducted, and a flow diagram illustrating the study selection process is provided (Fig. 3).

A systematic literature search was conducted to identify relevant peer-reviewed studies published between 2014 and January 2025. Relevant papers were identified through systematic search on Google Scholar and PubMed. Studies from peer-reviewed journals, conference proceedings, and peer-reviewed book chapters were considered. Keywords used included “SOZ,” “localization,” “classification,” “identification,” “machine learning,” and “iEEG.” A total of 57 articles were identified, of which 28 met the inclusion criteria after full-text screening. Studies addressing broader topics such as epileptic focus localization, focal vs. non-focal seizure classification, and epileptic biomarker classification were excluded, as these differ from the direct identification of SOZ. While these broader classifications can serve as a preliminary step in filtering data for further analysis, they do not equate to SOZ detection and are, therefore, beyond the scope of this review. We restricted our selection to studies that employed machine learning techniques and used iEEG data as the primary source. Non-invasive methods such as scalp EEG, neuroimaging, and traditional statistical approaches without machine learning were excluded. Key information, such as dataset size, features and biomarkers used, machine learning model, validation methods, performance, method strengths, and limitations, was extracted and thematically analyzed based on learning paradigms and clinical relevance. To explore performance trends, we conducted a comparative analysis focusing on accuracy and sensitivity across different learning paradigms and sample sizes. We also considered validation methods to assess generalizability.

There is an ongoing debate regarding whether only ictal data should be referred to as SOZ, while interictal data may better represent the epileptogenic zone (EZ). However, the terms are nearly identical when accurately localized. The distinction between SOZ and EZ is complex, even for clinicians. EZ is a theoretical concept representing the brain region responsible for generating and sustaining seizures, inferred from diagnostic tests, while SOZ is the specific location where seizures are observed on iEEG. The accuracy of both depends on electrode placement.

## Machine learning in SOZ localization

**Fig. 4:**
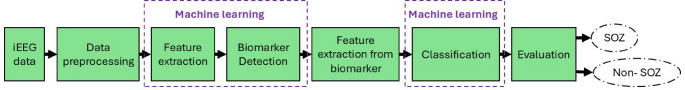
Block diagram illustrating common pipelines for automatic SOZ identification. First detects epilepsy biomarkers from iEEG data, followed by SOZ classification using biomarker features. Machine learning techniques are typically applied in the stages of feature extraction, biomarker detection, and SOZ classification

**Fig. 5:**
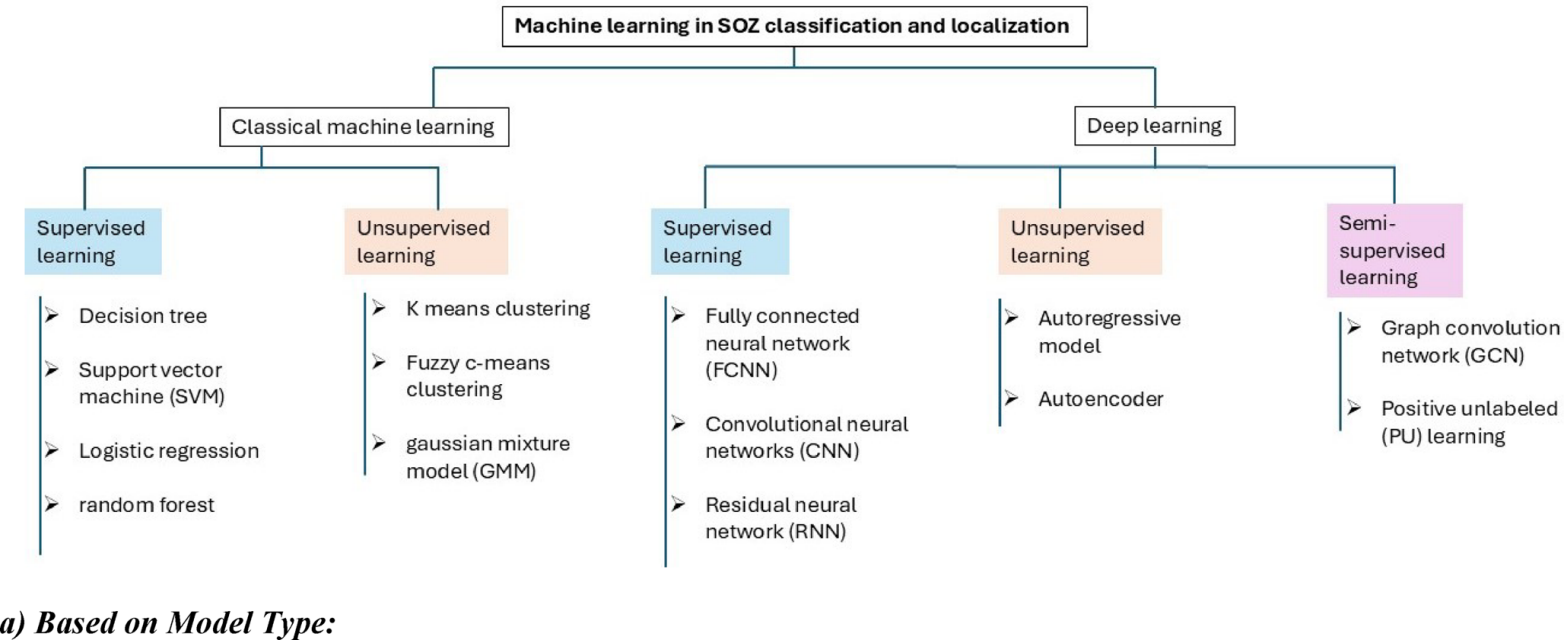
Overview of machine learning based classification methods for SOZ localization

Automatic identification of the SOZ typically follows a systematic pipeline, generally consisting of several stages: preprocessing, feature extraction, classification, and evaluation [53]. Throughout this pipeline, raw iEEG data undergoes preprocessing, followed by feature extraction. These features are then used for the detection of epilepsy-related biomarkers, often facilitated by machine learning models. Once biomarkers are identified, additional features are extracted—either statistically or automatically—for classification. Machine learning is again employed to classify these features as belonging to the seizure onset zone (SOZ) or non-SOZ. As shown in Fig. 4, machine learning plays a central role in key steps. To enable a more structured discussion, we classified the existing models based on their underlying technical approaches and discussed them. This categorization is presented as a tree structure for better visualization, as shown in Fig. 5.

- ***Based on model type***:

*i)Classical Machine Learning Models*: Classical machine learning models are generally more straightforward, have lower computational complexity, and often have well-defined mathematical foundations [54]. They usually rely on manual feature engineering, where relevant features from raw data are extracted before forwarding it to the model. Classical machine learning algorithms have played a significant role in SOZ localization. Techniques such as support vector machines (SVM) [62], decision trees [61], logistic regression [63], random forests [64], K-means clustering [65], fuzzy c-means [66], and Gaussian mixture models (GMM) [67] have been widely adopted across multiple studies. These models demonstrated competitive accuracy while maintaining lower computational demands. For example:

- SVMs were the most frequently used model [31, 35, 45, 48, 49, 52, 55, 56], achieving SOZ classification accuracies ranging from 73 to 94%, depending on feature types, datasets used, and validation type.
- Random forests, applied in [50, 58], showed robustness and strong generalizability in identifying SOZ with high sensitivity (~ 85%) in identifying SOZ electrodes.
- Logistic regression models, while simpler, provided interpretability and reasonably high performance (e.g., accuracy above 80% accuracy in [37, 51, 57]) when feature dimensionality was low.
- Fundamental model like decision tree showed good performance in detecting array of SOZ electrodes [40].
- K-means clustering [32, 42, 59] and GMMs [43] have been proven capable of distinguishing SOZ from non-SOZ by clustering iEEG signal features, especially with smaller datasets (accuracy up to 96.2%).
- Fuzzy c-means clustering [60] performed well in involving overlapping feature distributions and noisy iEEG signals.

*ii)Deep Learning Models*: Deep learning models, composed of multiple interconnected layers, can automatically extract relevant features from raw data, eliminating the need for manual feature engineering [54]. This capability makes them well-suited for SOZ localization, where raw time series or time-frequency representations can be directly input, enabling accurate and efficient identification of SOZ regions. In recent SOZ localization studies, deep learning models such as fully connected neural networks (FCNN), convolutional neural networks (CNN), residual neural networks (RNN) [70], graph convolution network (GCN) [71], and autoencoder based model in [72] are more prevalent due to their ability to extract complex features automatically:

- CNNs reached classification accuracy as high as 88% [35], enabling feature extraction from both iEEG signal [33, 34, 52] and spatiotemporal image data [35].
- The RNN model in [33] captured temporal dynamics of iEEG data to localize SOZ lobe.
- GCN in [68] integrated the dynamic and static characteristics of epileptic networks while autoencoder based model in [36] performed unsupervised feature extraction, enabling a semi-supervised GCN to generalize better on limited labeled data.

b. *** Based on learning paradigm***:

*i)Supervised Learning*: Supervised learning involves training models using labeled data to identify patterns that map inputs to correct outputs. In SOZ classification and localization, supervised models are widely adopted with labeled data where SOZ and non-SOZ regions, such as SOZ and non-SOZ electrodes, are pre-identified by experts. Standard supervised models used for SOZ localization include classification algorithms such as decision trees [40], SVM [31, 35, 45, 48, 49, 52, 55, 56], logistic regression [37, 51, 57], and random forests [50, 58], as well as advanced deep learning architectures like CNNs [33–35, 52], FCNNs [35, 49] and RNNs [33]. By leveraging complex patterns in iEEG data, supervised learning facilitates objective and data-driven identification of SOZ regions, uncovering relationships that might not be detectable through manual analysis. Supervised learning algorithms in these studies mostly require large, annotated datasets, which pose a significant challenge in practice.

*ii)Unsupervised Learning*: Unsupervised learning involves training models on unlabeled data to identify hidden patterns, clusters, or structures within the data without predefined outputs. Although applied in fewer studies, unsupervised learning explored and grouped iEEG features or biomarkers into clusters corresponding to SOZ and non-SOZ regions without explicit data definition. K-means clustering was used in studies such as [32, 42, 59] showing enhanced performance in separating SOZ electrode cluster from non-SOZ, despite a small cohort. GMMs with k-medoids [43] and fuzzy c-means clustering [60] achieved high specificity (up to 97.4%) but faced limitations such as class imbalance. Threshold based methods [69, 73] offered simple, annotation-free tools with promising sensitivity. Unsupervised feature extraction through attention mechanism based autoencoders [36] was applied in SOZ analysis and feature discovery. These techniques aided in feature grouping and initial SOZ identification, however, they generally required expert validation and were less suited for direct clinical use.

*iii)Semi-Supervised Learning*: Semi-supervised learning combines labeled and unlabeled data for improved generalization. Such approaches have recently emerged as promising tools for SOZ localization, particularly in settings with limited annotated data. For example, a GCN model [68] was employed in interictal HFO and seizure propagation, achieving high sensitivity (90.1%). Similarly, Zhao et al. [35] utilized a positive-unlabeled (PU) learning strategy on frequency band features, reaching 76.9% accuracy with minimal labeled data. These methods demonstrate the potential of learning from partially labeled datasets; however, challenges such as poor generalizability and false SOZ detections remain.

Tables 1 and 2 highlight the advancements in machine learning based SOZ localization from 2014 to 2025. Table 1 explains supervised learning approaches, while Table 2 focuses on unsupervised and semi-supervised learning methods. Both tables include the number of patients studied, iEEG recording period (ictal/interictal), machine learning models, feature types, performance metrics, strengths, and limitations of each approach. Performance is evaluated using accuracy, sensitivity, specificity, precision, and area under curve (AUC). However, not all studies reported all these metrics, so we included only those found in the reviewed studies. Analysis of these tables reveals that supervised learning remains the most commonly utilized approach among the learning paradigms, with SVMs frequently chosen as the preferred classification model due to their capability to tackle both linear and non-linear features. In many studies, HFOs have emerged as a widely used biomarker for SOZ localization. Over the years, researchers’ preference for iEEG recordings has gradually shifted from ictal to interictal periods. This shift is probably driven by the ease of obtaining interictal data and advancements in detecting relevant biomarkers during interictal periods. Additionally, there has been a noticeable trend of transitioning from classical machine learning-based methods to deep learning approaches, driven by their ability to handle complex patterns and larger datasets more effectively over the years.

**Fig. 6:**
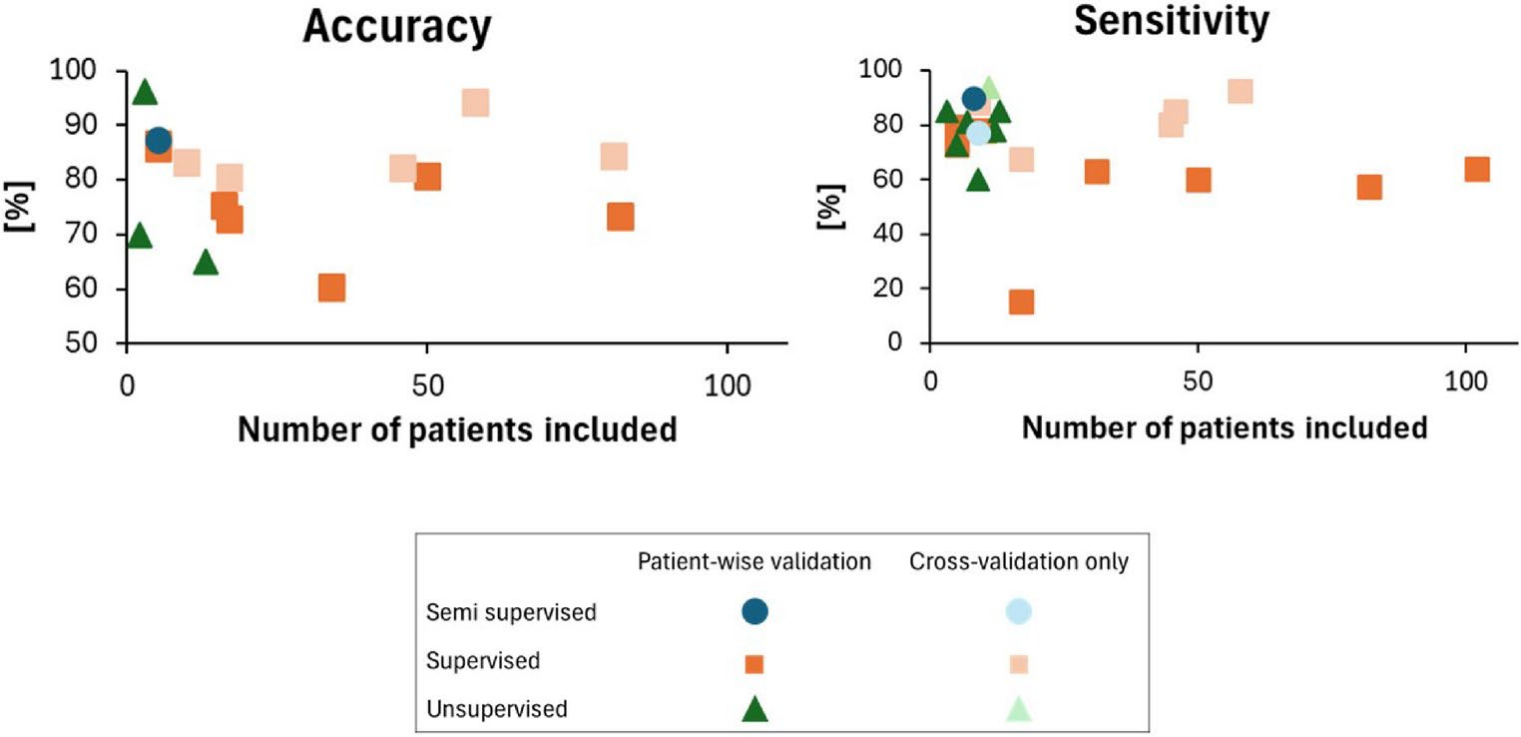
Trends in accuracy (left) and sensitivity (right) across different machine learning and validation methods for SOZ localization, shown relative to patient sample size

Using the data summarized in Tables 1 and 2, we conducted a trend analysis presented in Fig. 6, focusing on two performance metrics—accuracy and sensitivity—in relation to the learning paradigm (supervised, unsupervised, semi-supervised) and the number of patients. The primary emphasis was placed on variations across different learning paradigms. We selected accuracy and sensitivity as performance metrics for this analysis, as they best represent SOZ localization performance. Accuracy reflects the overall model performance for both SOZ and non-SOZ electrodes, while sensitivity specifically highlights the model’s effectiveness in identifying SOZ electrodes. Our findings indicate that studies with smaller sample sizes generally report higher accuracy and sensitivity, while studies with larger sample sizes tend to have lower performance. This trend suggests that high-performance results may be easier to achieve in studies with limited data but may not generalize well. Supervised learning is proven to be the dominant approach, particularly in studies with larger sample sizes. Unsupervised and semi-supervised methods appear mainly in smaller datasets and show varied performance. Additionally, differences in validation methods significantly impacted reported performance, with patient-wise validation consistently yielding lower performance compared to cross-validation studies. This underscores the importance of patient-wise validation and highlights challenges in generalization across different patients in current research trends.

**Table 1:** Supervised machine learning solutions for iEEG data based SOZ localization

Author (year of publication)	Patient number	Recording period	Biomarker	Machine learning algorithm	Performance	Strength	Limitations
Ellenrieder et al. (2016) [57]	45	Interictal	HFO rate, amplitude, frequency, duration	Logistic regression	Sen- 80% Spe-65%	Explored the differences between physiological and pathological HFOs	The evaluation was done only through cross-validation and lacks patient wise evaluation
Elahian et al. (2017) [51]	10	Preictal and ictal	PAC value	Logistic regression	Acc- 83% Pre- 90%	Low computational time and effective	The evaluation is done through cross-validation and lacks patient wise evaluation
Varatharajah et al. (2018) [45]	82	Interictal	HFO rate, IED rate, PAC rate	SVM	Acc- 73.3% Sen- 57.4% Spe- 79.5%	Experimented on a large dataset and proved the efficiency of multiple biomarkers	Comparatively lower performance
Grinenko et al. (2018) [31]	17	Ictal	Time-frequency pattern	SVM	Acc- 72.9% Sen- 15.3% Spe- 99.2%	Defined a simple fingerprint for SOZ localization	Poor positive class (SOZ) identification
Cimbalnik et al. (2018) [55]	31	Interictal	HFO rate, amplitude, frequency, and duration	SVM	AUC– 0.685 Sen- 63.9% Spe- 73.7%	Comparison of pathological vs. physiological HFOs with large patient cohort	Discriminative power of the model is moderate as well as reliance on manually extracted features
Lai et al. (2020) [56]	5	Interictal	Several HFO based features	SVM	Acc- 86.6% Sen- 73% Spe- 94.1%	Systematic use of channel-wise HFO features rather than event-wise	Small sample size and not validated with surgical outcomes
Besheli et al. (2022) [58]	16	Interictal	HFO rate	Random forest	Acc- 75.3%	Efficient and robust	The surgical outcomes of the patients are not confirmed
Craley et al. (2022) [33]	34	Ictal	Event related feature and seizure tracking	CNN, RNN and cross entropy	Acc- 60.5%	SOZ localization without any biomarker	SOZ localization was not electrode wise, rather lateralization and lobe wise.
Johnson et al. (2023) [34]	10	Ictal	--	1 dimensional CNN	Sen- 78.1% Spe- 74.6%	Can classify SOZ directly from iEEG-derived evoked responses	Large amount of training data required
Johnson et al. (2023) [48]	81	Interictal	Causal connectivity	SVM	Acc-84.4%	Provided valuable insights into the brain network dynamics	Lack of control groups and patient specific evaluation
Zhao et al. (2023) [35]	9	Interictal	Different frequency band entropies and STFT	SVM, FCNN, and CNN	CNN (Best Model) Acc- 88.1%	Explored potential of several frequency band features; achieved good performance in a semi supervised setup	Cannot be used in a patient-independent setting
Miao et al. (2023) [52]	7	Ictal and interictal	PAC features quantified by mean vector length modulation index	SVM, LightGBM, CNN	AUC- 0.76	Compared several machine learning approaches in SOZ localization	Imbalanced dataset affected SOZ detection
Balaji et al. (2024) [49]	58	Interictal	Causal connectivity	FCNN and SVM	Best Model = SVM Acc- 94.1% Sen- 92.3% Spe- 94.3%	Robust model exploring a large dataset	Lacks patient specific evaluation
Broti et al. (2024) [40]	5	Interictal	HFO density	Decision tree	Acc- 87.5%	Good performance from few data	The classification is electrode array wise, rather than electrode wise
Yan et al. (2024) [36]	17	Interictal	Temporal and spatial features extracted with autoencoder	GCN	Acc- 80.46% Sen- 67.31% Pre- 66.04%	Superior performance integrating the dynamic and static characteristics of epileptic networks	Lacks patient specific evaluation
Klimes et al. (2024) [37]	50	Interictal	univariate, bivariate, and event-related features of several frequency band	Logistic regression	Acc- 80.7% Sen- 60.1% Spe- 85.3%	Proved that lower frequency alone can be a good feature in SOZ localization	Poor sensitivity, probably caused by false positives in ground truth.
Chen et al. (2025) [50]	46	Interictal	Causal influence index derived from Phase transfer entropy	Random forest	Acc- 82.2% Sen- 85% Spe- 79.7%	Exhibits strong generalizability in identifying SOZ	Lacks patient specific evaluation

Acc = Accuracy, Sen = Sensitivity, Spe = Specificity, Pre = Precision, AUC = Area under curve, SVM = Support vector machine, LightGBM = Light gradient boosting machine, CNN = Convolutional neural network, RNN = Recurrent neural network, FCNN = Fully connected neural network, GCN = Graph convolution network, -- = not available

**Table 2:** Unsupervised and semi supervised machine learning solutions for iEEG data based SOZ localization

Author (year of publication)	Patient number	Recording period	Biomarker	Machine learning algorithm	Performance	Strength	Limitations
Malinowska et al.(2014) [42]	33	Preictal, Interictal and ictal	HFO rate	K means clustering	--	Investigated importance of ictal and non-ictal HFOs	SOZ localization performance not evaluated
Geertsema et al.(2015) [30]	9	Ictal	Nonlinear iEEG features	Autoregressive model residual variation	Sen- 60% Spe-70%	Time efficient and unsupervised	Performance is comparatively low
Liu et al. (2016) [43]	7	Preictal	HFO frequency, sub band power ratio, spectral entropy	GMM with k-medoids	Sen- 81% Spe- 96%	Robust and Efficient	High imbalance in SOZ and Non-SOZ channel number
Malladi et al. (2016) [47]	5	Preictal, ictal, interictal and post ictal	Causal Connectivity	Data driven clustering	--	Determined SOZ directly from causal interactions	Comparatively poor performance
Murphy et al(2017) [73]	2	Interictal	HFO rate	Threshold based index measurement	Acc- 70%	Simple unsupervised approach	Due to small patient size, the approach is not proven to be generalized
Quitadamo et al. (2018) [59]	12	Interictal	HFO rate	K means clustering	Sen- 77.9% Spe − 71.4%	Provides an easy-to-use tool for HFO classification and SOZ localization from HFO rate	Performance for each patient is not consistent
Charupanit et al. (2020) [69]	11	Interictal	HFO amplitude	Threshold based index measurement	Sen-93.7%	Excellent performance without annotation	The number of electrodes per patient was small. Only cross-validation evaluation was performed.
Wan et al. (2020) [60]	5	Interictal	HFO features (mean singular value, line length, power ratio, spectral centroid)	Fuzzy c means clustering	Sen- 72.7% Spe- 97.4%	Eliminates noise and finds cluster through learning	Always classify some electrodes as SOZ for each patient even when the patient does not require surgery
Xiao et al. (2021) [32]	3	Interictal	Adaptive high frequency epileptogenicity index	K means clustering	Acc- 96.2% Sen- 85.1%	Very precise SOZ localization	Small patient population
Liu et al. (2022) [68]	8	Interictal	HFO and seizure propagation	GCN (semi supervised)	Sen- 90.1% Pre- 44.5%	Small amount of annotated data required	Many false SOZ electrode detection
Zhao et al. (2023) [35]	9	Interictal	Different frequency band entropies and STFT	PU (semi supervised)	Acc = 76.9%	Explored potential of several frequency band features; achieved good performance with very few data	Cannot be used in a patient-independent setting

Acc = Accuracy, Sen = Sensitivity, Spe = Specificity, Pre = Precision, GMM = Gaussian mixed model, GCN = Graph convolution network, PU = positive unlabeled– = not available

## Benefits of using machine learning

Machine learning offers a range of advantages for SOZ localization by enhancing efficiency, accuracy, and objectivity. From extracting discriminative and independent features from iEEG data to identifying the structure in which the unknown parameters lie, machine learning has proven to be a versatile and transformative tool. Below, we highlight the key benefits, supported by examples from the literature:

- ***Complex pattern recognition capability***.

Machine learning algorithms excel at identifying complex patterns in high-dimensional iEEG data. For instance, Zhao et al. [35] applied CNNs to frequency band entropies and STFT features, successfully distinguishing SOZ from non-SOZ. Similarly, Yan et al. [36] utilized GCN to integrate spatial and temporal features, achieving high accuracy, thus highlighting the capability of these models to detect subtle biomarkers or SOZ signatures that may not be visually apparent [4, 11, 40–45].

b) ***Finding linear and non-linear relationships***.

Algorithms like SVMs and deep neural networks can capture both linear and nonlinear relationships among features. Miao et al. [52] compared SVM, CNN, and LightGBM models using PAC features and reported meaningful results, demonstrating that machine learning can model complex dependencies among biomarkers. Balaji et al. [49] also achieved high classification performance (SVM accuracy of 94.1%) by modeling causal connectivity features, showcasing the benefit of learning nonlinear interactions among network dynamics.

c) ***Robustness to randomness in data***.

iEEG recordings often contain noise or patient-specific variability. Machine learning models are adept at filtering out irrelevant patterns. For example, using fuzzy c-means clustering on HFO features, high specificity (97.4%) was achieved despite the presence of noise and overlapping signal characteristics [60]. Johnson et al. [34] demonstrated CNN based models that reliably classified SOZ from evoked responses, even with variable input data.

d) ***Validation of hypotheses***.

Machine learning enables systematic testing of biomarkers. Logistic regression was utilized to prove the usefulness of lower frequency in SOZ localization in [37] as well as in [57] to explore the differences between physiological and pathological HFOs. Liu et al. [43] validated spectral entropy and HFO frequency features using a GMM. Using unsupervised clustering, Malinowska et al. [42] investigated importance of ictal and non-ictal HFOs.

e) ***Minimizing subjectivity and labor***.

Manual annotation of SOZ regions is time-consuming and subjective, while semi-supervised and unsupervised models can solve this by automating classification based on data-driven patterns. For example, semi-supervised models like Liu et al. [68] and Zhao et al. [35] required minimal labeled data, illustrating reduced reliance on manual annotation. Unsupervised approaches achieved good performance without needing any manual annotation at all [32, 43, 59].

f) ***Time efficiency and real-time potential***.

Once trained, models can process large amounts of data in a fraction of the time required for manual analysis. Additionally, advancements in computational power and optimization techniques allow for near real-time analysis, enabling clinicians to use these tools during surgery or in continuous monitoring scenarios [30, 43, 58].

g) ***Scalability and efficiency***.

Machine learning is highly scalable and can efficiently analyze data from multiple patients or large electrode arrays. Varatharajah et al. [45] used an SVM on a dataset of 82 patients and demonstrated the efficiency of combined biomarkers such as HFO, IED, and PAC rates. Similarly, Klimes et al. [37] used simple models such as logistic regression with 50 patient data to achieve robust performance across large data scales.

The reviewed studies provide empirical evidence that machine learning is not only theoretically advantageous but also practically effective in improving SOZ localization. The integration of machine learning models has enabled better handling in iEEG analysis—thereby supporting efficient clinical decision-making.

## Limitations and challenges

Machine learning has shown promise in SOZ localization. However, some critical issues remain that hinder this field of research. While reviewing the existing works, we found the following challenges.

- ***Lack of absolute confidence in SOZ identification***.

**Fig. 7:**
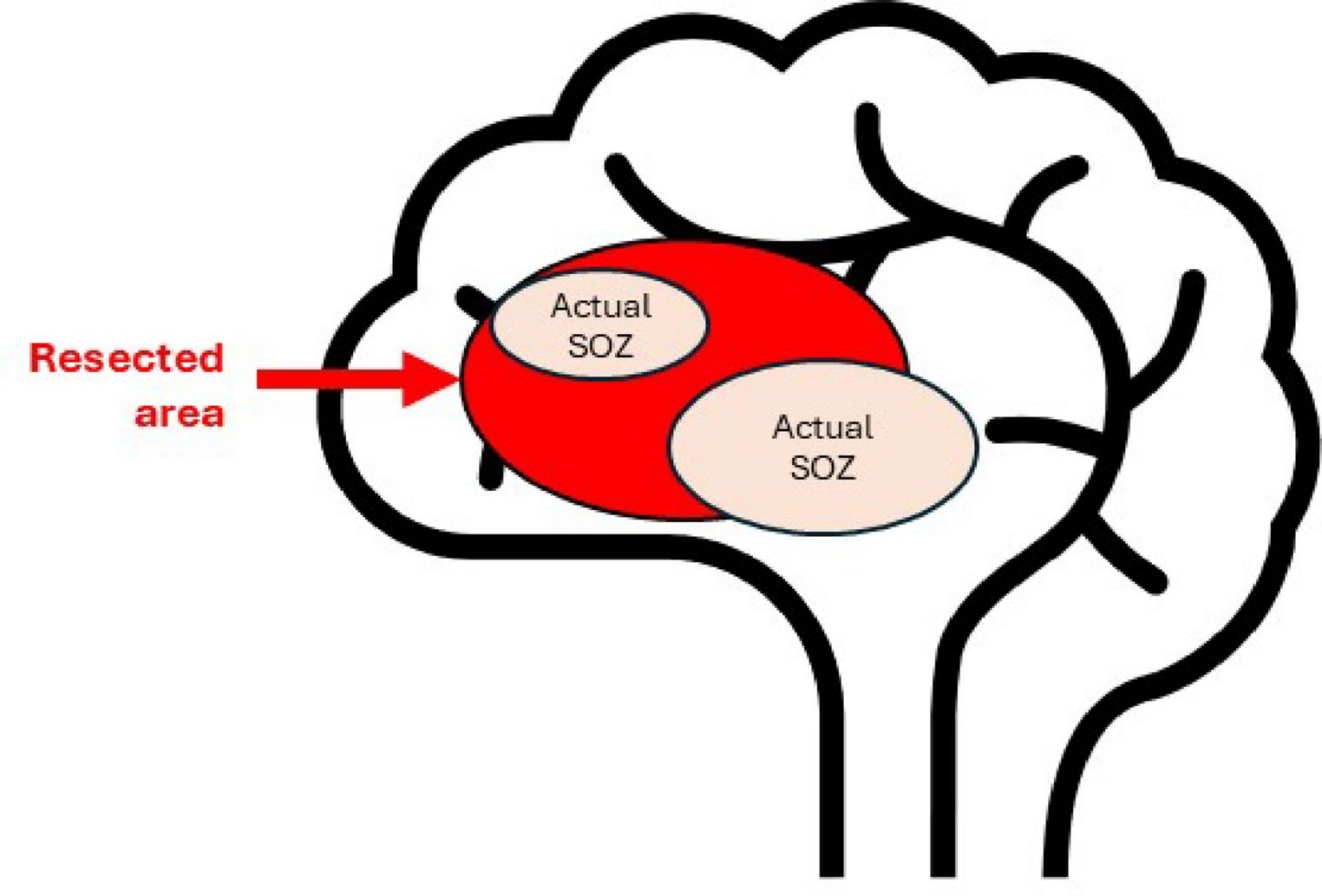
Resected area and SOZ; While entire resected area is usually labeled as SOZ for machine learning models, in reality, a fraction of resected areas might be actual SOZ

The lack of a universally accepted definition of SOZ presents a major challenge for both clinical practice and machine learning applications. Even expert clinicians rely on subjective interpretation of complex iEEG patterns and supporting clinical data. Surgical outcomes, often used to validate SOZ identification, are not always successfully suggesting possible inaccuracies in the labeled SOZ. Resection areas may extend beyond or fall short of the true SOZ [19, 25] (Fig. 7), potentially inflating false positives or negatives. These inconsistencies contribute to lower model sensitivity and introduce noise into training and evaluation, hindering reliable performance. Advanced unsupervised approaches such as deep clustering [88] and NLP techniques like word2vec [75, 89] may help refine SOZ definitions and improve model outcomes.

b) ***Very few public datasets***.

Validating machine learning models for SOZ localization is hindered by the scarcity of publicly available datasets and lack of standardized benchmarks. SOZ data, typically collected during epilepsy surgery evaluations [25], is often proprietary due to privacy and ethical concerns. As a result, only a few datasets—such as the Bern-Barcelona [76] and JHH [77] datasets—are publicly available, limiting model development, reproducibility, and generalizability. Addressing this challenge requires data-sharing initiatives with anonymized datasets and the creation of benchmarks. Additionally, techniques like generative adversarial networks (GANs) [78] and transfer learning [4, 79] can help mitigate data scarcity.

c) ***Lack of a Golden Biomarker***.

Unlike many medical conditions where a specific biomarker can reliably indicate the presence of disease, epilepsy and SOZ localization lack a definitive, gold-standard biomarker [5]. The identification of the SOZ relies on analyzing a complex combination of electrophysiological, structural, and functional features, none of which alone are sufficient for accurate localization. This makes it challenging to design machine learning models, as these models often require clear target variables for training and validation. Multi-modal classification is a promising approach to overcome these issues. Combining several biomarker data sets with electrophysiological, structural, and functional data to comprehensively represent SOZ characteristics can improve performance largely [45, 80].

d) ***Coarse subdivision of electrodes***.

Clinical hypotheses guide electrode placement in invasive monitoring and are often sparse, covering only suspected areas of interest rather than the entire brain. This spatial sparsity can lead to coarse-grained data, where subtle differences in SOZ activity may be missed. For machine learning models, this limitation reduces the resolution of input features, hindering the ability to accurately identify patterns associated with SOZ. Advanced high-density grids or SEEG probes can increase spatial coverage and resolution, capturing subtle SOZ activity across a broader brain region. Integration with non-invasive modalities can further improve performance.

e) ***Difficulty in achieving generalized performance***.

Accurate SOZ localization remains difficult, even for advanced machine learning models, due to biological variability and heterogeneity in seizure patterns among patients. Small datasets and a lack of standardized evaluation metrics further hinder model performance. While many models perform well in controlled settings, they often fail to generalize to real-world clinical scenarios [35, 36, 48–50, 57]. Expanding datasets to include diverse patient populations across age groups and pathology types is crucial for improving generalizability and clinical utility.

f) ***Limited application of deep learning***.

Despite deep learning’s success in many fields, its use in SOZ localization remains limited. iEEG datasets are typically small and high-dimensional, making deep learning models prone to overfitting without sufficient labeled data. Moreover, the lack of interpretability poses challenges for clinical adoption, where transparency is essential. As a result, researchers have been cautious in applying deep learning techniques to SOZ localization. However, emerging approaches—such as graph neural networks, transformers, attention mechanisms, and self-supervised learning—offer promising solutions for handling complex, structured data. Explainability tools like saliency maps [81], Shapley additive explanations SHAP [82], and attention based visualizations [83] can enhance model transparency, supporting clinical validation.

## Discussion

Over the past decade, machine learning has significantly advanced SOZ localization in epilepsy, evolving from traditional supervised methods to deep learning approaches. This narrative review synthesizes research on iEEG based SOZ detection from 2014 to 2025, covering a wide range of methodologies, including supervised, unsupervised, and semi-supervised models across varied sample sizes. It highlights key progress in improving accuracy, efficiency, and scalability, while also addressing current limitations and the ongoing need for effective epilepsy treatments. By examining methodologies, findings, and innovations, we offer insights into emerging trends, clinical relevance, and future directions.

Machine learning has transformed SOZ localization, offering a more objective and efficient alternative to traditional methods. Supervised learning remains dominant due to its ability to model both linear and non-linear patterns in complex iEEG data. A growing trend is the use of interictal data, which is easier to acquire than ictal data. More recently, deep learning has gained traction for its superior ability to handle large, complex datasets and capture intricate iEEG patterns. Preliminary analysis shows that studies with smaller sample sizes often report higher accuracy and sensitivity, likely due to reduced complexity and noise. However, performance tends to decline with larger, more diverse datasets, highlighting the need for models that generalize well. This points to a trade-off between performance and scalability. Additionally, variations across validation methods underscore the importance of patient-wise validation, which better reflects a model’s ability to generalize across individuals and should be prioritized for clinical relevance. Deep learning is proving to be a powerful tool for SOZ localization due to its ability to capture complex spatiotemporal patterns and reduce subjective interpretation of iEEG signals.

This review highlights key challenges in achieving full automation of SOZ localization, including ambiguity in SOZ annotation, limited benchmark datasets, poor generalizability across patient populations, and underutilization of advanced deep learning methods. Future progress relies on developing large, diverse, multi-institutional datasets and promoting data-sharing initiatives to support standardization and reproducibility. Advances in unsupervised and semi-supervised methods, such as deep clustering [88] can offer data-driven insights into SOZ characterization. Natural language processing methods can help clinicians model relationships between SOZ-related features [89]. Integrating multimodal data (e.g., iEEG, MEG, fMRI) and multiple biomarkers can improve accuracy and robustness [45, 80]. Several machine learning approaches have demonstrated the benefits of multimodality in clinical studies of epilepsy [84–86, 90]. Explainable artificial intelligence techniques can further enhance model interpretability and clinical trust [81–83]. Additionally, wearable technologies could enable continuous, non-invasive SOZ tracking [87]. Collaboration among researchers, clinicians, and engineers is essential to overcoming current limitations and advancing machine learning in epilepsy care.

While the review aimed for a comprehensive synthesis, the rapid pace of research in this field can imply that some very recent developments may have been omitted during the review. Furthermore, we included studies that used interictal data, which may technically not be applicable for SOZ. Finally, the diversity in reported evaluation metrics and experimental setups among studies complicates direct comparisons and the extraction of universal trends. A systematic review of existing peer-reviewed articles and experimental validation on several open-access datasets might help the new researchers to a great extent.

## Conclusion

This article explored SOZ localization from both clinical and engineering perspectives, taking a step toward more efficient automated epilepsy diagnosis and treatment planning. Findings revealed that developing robust, efficient, and generalized machine learning-based methods for SOZ localization is a promising direction in epilepsy research. However, developing a trustworthy and clinically reliable system remains a significant challenge. Therefore, future machine learning approaches for SOZ localization must consider clinical needs, including interpretability and ease of integration into clinical workflows. Additionally, fostering collaboration between engineering and medical sectors is crucial for developing innovative machine learning approaches. The review findings can help advance diagnostic accuracy, optimize treatment strategies, and ultimately enhance the quality of life for patients with epilepsy.
